# Paper 3: Selecting rapid review methods for complex questions related to health policy and system issues

**DOI:** 10.1186/s13643-021-01834-y

**Published:** 2021-10-30

**Authors:** Michael G. Wilson, Sandy Oliver, G. J. Melendez-Torres, John N. Lavis, Kerry Waddell, Kelly Dickson

**Affiliations:** 1 McMaster Health Forum, McMaster University, Hamilton, Canada; 2 Department of Health Evidence and Impact, McMaster University, Hamilton, Canada; 3 Centre for Health Economics and Policy Analysis, McMaster University, Hamilton, Canada; 4 EPPI-Centre, University College London, London, UK; 5 Africa Centre for Evidence, University of Johannesburg, Johannesburg, South Africa; 6 College of Medicine and Health, University of Exeter, Exeter, UK; 7 Department of Political Science, McMaster University, Hamilton, Canada

## Abstract

Approaches for rapid reviews that focus on streamlining systematic review methods are not always suitable for exploring complex policy questions, as developing and testing theories to explain these complexities requires configuring diverse qualitative, quantitative, and mixed methods studies. Our objective was therefore to provide a guide to selecting approaches for rapidly (i.e., within days to months) addressing complex questions related to health policy and system issues.

We provide a two-stage, transdisciplinary collaborative process to select a rapid review approach to address complex policy questions, which consists of scoping the breadth and depth of the literature and then selecting an optimal approach to synthesis. The first stage (scoping the literature) begins with a discussion with the stakeholders requesting evidence to identify and refine the question for the review, which is then used to conduct preliminary searches and conceptually map the documents identified. In the second stage (selection of an optimal approach), further stakeholder consultation is required to refine and tailor the question and approach to identifying relevant documents to include. The approach to synthesizing the included documents is then guided by the final question, the breadth and depth of the literature, and the time available and can include a static or evolving conceptual framework to code and analyze a range of evidence. For areas already covered extensively by existing systematic reviews, the focus can be on summarizing and integrating the review findings, resynthesizing the primary studies, or updating the search and reanalyzing one or more of the systematic reviews.

The choice of approaches for conducting rapid reviews is intertwined with decisions about how to manage projects, the amount of work to be done, and the knowledge already available, and our guide offers support to help make these strategic decisions.

**Supplementary Information:**

The online version contains supplementary material available at 10.1186/s13643-021-01834-y.

## Background

The literature about rapid reviews has grown substantially in recent years both in terms of approaches for accelerating and streamlining the conduct of systematic reviews (e.g., for searching, screening, extracting data, quality appraisal and synthesis of results) and with examples of rapid reviews that have been conducted using these approaches [1–11]. However, many of these approaches and examples of rapid reviews often adopt a narrow focus by focusing on a particular population, intervention, controls, and outcomes (i.e., the PICO framework).

Such approaches to conducting rapid reviews are amenable to many questions of effectiveness (e.g., whether a particular clinical intervention works for a specific population and outcome as compared to standard care). The types of issues that policymakers often have to grapple with as part of efforts to strengthen and reform health systems through often complex policy and programmatic changes may require a broader spectrum of considerations that are not optimally addressed by streamlining a traditional systematic review approach. For example, a PICO framework approach does not allow for the exploration of complexities that arise from “variations within populations or interventions, or about the mechanisms of action or causal pathways through to mediate outcomes, other contextual factors that might similarly moderate outcomes, or how and when these mechanism and elements interact.” [12]. In addition to this complex array of factors, policymakers also need to use a different types of evidence and data to clarify what is driving a particular policy issue [13], identify and frame policy options (including understanding benefits, harms, costs, adaptation that needs to be made one or more interventions to ensure it works locally and the views and experiences of stakeholders that might determine its acceptability) [14], and determine how one or more policy options can be best implemented at a system level [14]. Generating a synthesis of such data and evidence also often requires a mix of (1) policy analysis (i.e., a synthesis of best-available evidence and insights from key informants), (2) systems analysis (i.e., an analysis of policy documents, websites and insights from key informants about how systems work and how to do things differently), and (3) political analysis (i.e., an analysis of policy documents, websites and insights from key informants to identify factors that affect government agenda setting and policy choices) [15].

Moreover, given the need to respond to political priorities as they emerge, the timelines in which policymakers need to find and use research evidence to inform policy can vary from days, to weeks to months [8]. As a result, those working to support the use of research evidence in policy decision-making need to adjust their timelines for synthesizing evidence in order to respond before a given “window of opportunity” to inform pressing health-system issues closes. Therefore, our objective in this paper is to provide a guide for selecting approaches that can be used for policy-relevant rapid (i.e., within days to months) reviews.

### Main text

**Fig. 1:**
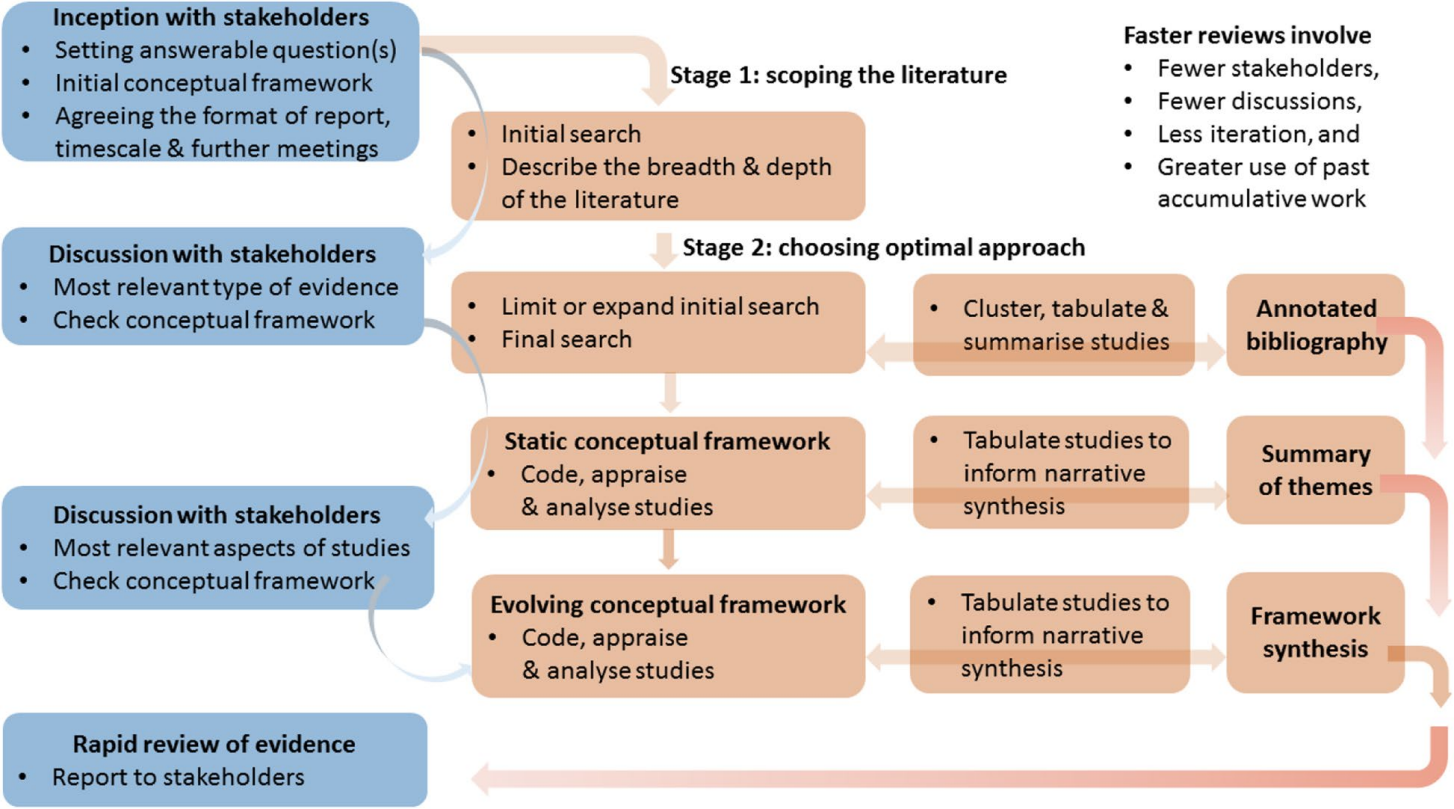
Overview of two-stage rapid review process (adapted with permission from Oliver et al.) [11]

Using insights from groups that conduct rapid reviews (including our own) that we identified from contributors to a recent guide for conducting rapid reviews to strengthen health policy and systems [16] and from our respective networks, we derived a set of considerations for conducting rapid reviews to address complex health policy and systems issues. We present this in a two-stage approach, which we depict in Fig. 1.

#### Stage 1—Consultation to develop the synthesis question

The first stage is focused on consultation and initial scoping of the breadth and depth of the literature to develop a focus and question that is feasible to address in the policy timeline provided. Consultations at this stage (and in the next stage) often require open discussion that draws on the strengths and expertise of the requestor and those conducting the synthesis. Indeed, engaging multiple stakeholders has been considered essential both for the membership of initiatives such as guideline development groups, but also for processes for developing new review protocols [11, 17]. Moreover, making the most of the input from such groups is easier when individuals who “wear multiple hats,” particularly knowledge brokers (i.e., individuals or organizations who are skilled in navigating the interface of policy and research) are engaged in the process [11, 18].

During such consultative processes, requestors need to provide clarity about what’s needed to inform the policy challenge they are addressing and the context for the question (e.g., why is a change being considered, who are the key actors and any political sensitivities in relation to the question) in order to help refine the question to be addressed. In particular, it should be clarified whether they are they looking for evidence to clarify a policy problem, options to address a problem, implementation considerations and/or monitoring and evaluation plans, and whether they are looking for one or more of a policy, systems, or political analysis that may require either using existing frameworks or deriving a new framework. Given that each of these decisions will have an impact on the ability to meet the timeline provided, this may require being open to refining the question to make it amenable to searching for and synthesizing evidence to meet the required deadline. For those conducting the synthesis, supporting an effective consultative process often means offering expertise in shaping researchable questions, as well as ensuring a systematic and transparent synthesis process while also being flexible and knowledgeable about the range of types of evidence and synthesis approaches that could be used in different timelines.

Following this initial consultation, the team leading the review will need to conduct an initial scoping of the literature available before judging the feasibility of what has been requested in the timeline provided. This can involve pilot searches in key database, scanning the search results and mapping (e.g., according to areas of interest and/or outcomes) what is found in order to establish the volume and type of literature that will likely need to be reviewed, which is then used to inform further consultations and refinement in the 2nd stage.

#### Stage 2—Refining the question to select an optimal approach for the question posed and the timeline provided

Following stage 1, additional consultation with the requestor (and, if needed, external stakeholders and experts in the area) is typically needed to further shape the focus of the review and question(s) and to decide on the optimal approach to conducting the review within the timeline provided. This set of consultations will ensure that a rapid *and* policy-relevant synthesis is produced in a way that is systematic and transparent in its approach. Given that the timeline provided by the requestor is the driving factor for producing a rapid review, we outline three broad approaches to conducting rapid reviews for complex questions related to health policy and system issues that can be used to meet three different policy timelines. We provide an overview of these timelines (days, weeks, or months) and approaches for each (annotated bibliography, thematic summary, and synthesis) along with illustrative examples of organizations that produce each type of review in Table 1. In addition, we provide further insight below about each of the synthesis approaches we identify, the sources of evidence that can be used and project management considerations for conducting rapid reviews of complex questions related to health policy and system issues. These are complemented by illustrative examples of reviews that have been conducted using each of the three broad types of methods that we outline in Table 1.

**Table 1:** Overview of approaches to rapid reviews according to three different policy goals and timelines

Timeline and goal	Synthesis approach to address a health policy or system issue^**a**^	Types of evidence to consider including	Project management considerations	Illustrative examples of groups that produce each type of rapid review
Days • e.g., to inform urgent internal policy discussions and/or management decisions	Profile of existing evidence • Uses a focused question and sub-questions to guide a targeted and rapid search (often using sources for pre-appraised evidence such as Health Systems Evidence) to enable efficient identification of the most relevant evidence • Reviews in this timeline typically only conduct a policy analysis^b^ (e.g., assessment of benefits, harms and/or costs of policy options), but systems analysis may be feasible depending on the scope. • A structured profile consisting of tables that map and summarize the identified literature (e.g., according to themes or domains of interest) along with a brief narrative summary of the types of documents found and their focus typically forms the final review.	• Evidence from systematic reviews o Overviews of systematic reviews o Systematic reviews • Evidence from primary studies o Not typically feasible to focus on primary studies given constrained time that limits ability to conduct and review comprehensive searches and synthesize findings • Other sources of evidence o Key informant interviews may be conducted if time permits to identify additional literature and insights about the topic (depends on the timeline and availability of key informants) o Not typically feasible to include other types of evidence (e.g., evidence from policy documents and websites) given the time required for hand searching	• Brief consultation with a small core transdisciplinary rapid review team before and in final stage of the review to efficiently brainstorm an approach to searching, how to organize findings and to conduct the final write-up. • Review best conducted with 1–2 reviewers sharing the work to enable rapid iterations of the review (e.g., to compare evidence needed with what has been identified) • A merit review process (i.e., where policymakers, stakeholders and/or researchers review the synthesis for policy relevance and scientific merit) is typically not feasible in this timeline.	• McMaster Health Forum (also conducts reviews in weeks or months) [15, 29] • Canadian Agency for Drugs and Technologies in Health (also conducts reviews in weeks or months) [20] • K2P Centre (also conducts reviews in weeks or months) [2]
Weeks • e.g., to inform public debates	Summary • May use a mix of policy, systems and political analysis • Review includes a summary of key findings in tabular format that may be organized by an existing thematic framework (e.g., by a typology of approaches and/or outcome domains) • Tables are typically accompanied by a brief narrative summary that highlights key findings and themes or a summary of whether and how a policy option has been used in a small number of jurisdictions (i.e., for a systems analysis) • A timeline of weeks typically does not allow for a true synthesis of findings, which would require a combination of or re-analysis of findings from the literature through quantitative methods or qualitative thematic or framework analysis	• Evidence from systematic reviews (same as above) • Evidence from primary studies o Targeted searches of a small number of highly relevant databases (searches are sometimes narrowed by country focus depending on the scope of the question) o A reanalysis of primary studies from systematic reviews (e.g., existing systematic reviews that address broad questions can provide a short-cut to identifying relevant studies for narrower rapid review question) • Other types of evidence o Key informant interviews can be conducted to identify additional literature and insights about the topic, but with more stakeholders than what is feasible in the previous row o Targeted hand searches for policy documents and websites to conduct a system and/or political analysis related to a small number of comparator jurisdictions	• Brief consultation with a small core transdisciplinary rapid review team before and in final stage of the review to efficiently brainstorm an approach to searching, how to organize findings and to conduct the final write-up. • Review may be best conducted with larger team that can apply standardized procedures to review search results and extract data from included documents • Some members of the review team can be deployed to conduct hand searches for policy documents and websites (e.g., to inform a targeted system analysis that includes a small number of comparator jurisdictions) • A merit review process can be completed within this timeframe in order to receive feedback from policymakers, stakeholders and researchers about policy relevance and scientific rigor.	• All examples from previous row • SURE [5, 30, 31] • EVIPNet Chile [3]
Months • e.g., to inform policy development cycles that have a longer timeline, but that cannot wait for a traditional full systematic review	Synthesis • Typically incorporates multiple types of analyses (e.g., policy, systems and political analysis) and applies an evolving framework to synthesize findings • With the longer timeline a synthesis may involve generating analyses from a broader policy domain and from across more jurisdictions for systems and policy analyses, as well as integrating (i.e., synthesizing) the findings from these analyses using an evolving framework that is generated based on emerging concepts from the literature and insights from key informants • An initial framework may come from existing theories as a “best fit” for analysis (e.g., when the review addresses a question that draws on a well-established literature with a specific discipline) or derived from consultations with the requestors and/or key informants to use for the analysis (e.g., when the review questions are transdisciplinary and an overarching framework is unlikely to exist)	• Evidence from systematic reviews (same as above) • Evidence from primary studies o Broader searches and in more databases o Update of systematic reviews (e.g., where a review on the same topic exists, but is out-of-date) o Reanalysis of primary studies from systematic reviews by applying inclusion criteria from the rapid review question to a previously conducted systematic review to generate a set of relevant studies to re-analyze but with a different or narrower focus (note that this could accompany an update a systematic review) • Other types of evidence o Key informant interviews, but with more stakeholders than what is feasible in the previous row o Broader hand searches for policy documents and websites to conduct a system and/or political analysis related to a larger number of comparator jurisdictions	• Brief consultation with a small core transdisciplinary rapid review team before and in final stage of the review to efficiently brainstorm an approach to searching, how to generate findings and to conduct the final write-up. • Review may be best conducted with a small review team in order to allow for more in-depth interpretation and iteration	• All examples from first row • EPPI-Centre • Evidence Check (Sax Institute) [22] • Evidence Alliance [32] • African Centre for Evidence

^a^As described earlier in the paper, questions may focus on one or more components of a typical policy-development cycle which includes clarifying a problem, identifying options to address a problem, identifying implementation considerations, and developing monitoring and evaluation plans

^b^ As described earlier in the paper, rapid reviews addressing complex policy questions often requires a mix of (1) policy analysis (i.e., a synthesis of best-available evidence and insights from key informants). (2) systems analysis (i.e., an analysis of policy documents, websites and insights from key informants about how systems work and how to do things differently), and (3) political analysis (i.e., an analysis of policy documents, websites and insights from key informants to identify factors that affect government agenda setting and policy choices) [15]

In terms of project management, some activities are required across each type of method while other activities may differ depending on the timeline and scope of the review. What is consistent across each type of review is the need for brief consultation with a small core transdisciplinary team before starting the review and in the final stages. As noted above, transdisciplinary teams are strengthened by engaging those who are engaged in many roles and/or who have extensive experience with navigating the interface of policy and research [11, 18]. Team-based consultations with such individuals before starting a review are invaluable for brainstorming an approach for searches and possible ways to organize the findings. Options include using a suitable existing framework throughout the review (a static framework) or starting with an orienting framework that can be refined over the course of the review (an evolving framework). In addition, the same team or stakeholders can review and refine the presentation of the findings and “fresh eyes” from team members that were not actively involved in the process of searching, reviewing and extracting data can help refine the organization of findings to ensure policy relevance.

Team size for conducting the review may need to vary depending on the type of review chosen. Team size for the relatively undemanding task of preparing an annotated bibliography (within days) may depend entirely on the number of studies identified. . For a synthesis of findings (within months), a smaller team of 1–2 reviewers working together over a period of months helps to facilitate more in-depth interpretation and iteration, which can be supported by periodic brainstorming meetings with a larger core transdisciplinary team. In contrast, producing a thematic summary within weeks may be best achieved with a “divide and conquer” approach with a larger team of reviewers that can apply standardized procedures to review search results, and extract data from included documents. This typically requires delegating tasks down to the lowest level of staff that can be trained to consistently execute tasks to a high standard. Moreover, for questions that require reviewing research evidence as well as incorporating other types of evidence (e.g., from policy documents and/or insights from key informants), some team members can be deployed specifically to those tasks while others focus on reviewing evidence.

#### Overview of rapid review approach 1—Profile of existing evidence (produced in days)

This approach is driven by very short policy timelines (typically when only a few days are available to conduct the review). Given this, a focused question to guide targeted searches for synthesized evidence from sources for pre-appraised evidence (e.g., Health Systems Evidence) is required. Typically, only a policy analysis is feasible to produce (i.e., an assessment of benefits, harms or costs of a policy option) and in a format for an annotated bibliography (e.g., tables that map and summarize identified literature). This can be accompanied with a brief description of the types of evidence found and their focus. Key examples of such include a review produced in three business days is a rapid synthesis produced by the McMaster Health Forum that provided key messages from three overviews of systematic reviews and 36 systematic reviews about the effects of homecare on improving health outcomes, client satisfaction, and health system sustainability [19]. In addition, the Canadian Agency for Drugs and Technologies in Health produces similar types of reviews in this timeline (e.g., in the form of reference lists and summaries of abstracts) [20]. While more challenging, a system or political analysis could also be feasible in this timeframe, depending on the nature of the request. For example, a rapid synthesis was produced in three business days to identify performance measures, indicators and targets to monitor and evaluate dementia strategies, which required identifying and review policy documents from hand searches of government websites [21].

#### Overview rapid review approach 2—Thematic summary (produced in weeks)

With a timeline of several weeks, a rapid review can produce a thematic summary of evidence based on a mix of policy, systems, and/or political analyses. Such reviews often draw on evidence from a range of sources including existing systematic reviews, primary studies (e.g., through a targeted search of small number of databases or a reanalysis of a subset of primary studies from existing systematic reviews that address a more focused or slightly different question posed for a rapid review), policy documents, and interviews with key informants who can provide additional insights and suggestions for literature that may not be found through database searches. The resulting product for a summary produced within several weeks often takes the form of a mix of tables that are organized using an existing thematic framework and an accompanying narrative that highlights key findings and themes or (in the case of system analysis) a summary of whether and how a policy option has been used in a small number of jurisdictions. However, a timeline of several weeks does not allow for a true synthesis, which requires a combination or re-analysis of findings using quantitative methods and/or qualitative methods thematic or framework analysis.

There are many examples of these types of rapid reviews. Examples that highlight a range of topics include a the rapid “evidence checks” that are prepared by the Sax Institute [22], a 10-week review of medical malpractice policies [23], and a summary of the use of and compensation for virtual-care services in primary care that was conducted in 6 weeks [24].

#### Rapid review approach 3—Synthesis (produced in months)

In instances where requestors are not in as big of hurry to receive the rapid review (e.g., when several months are provided to conduct the work), those conducting the review can generate multiple types of analyses (e.g., policy, system and political analyses using quantitative and/or qualitative methods), focus on a broader policy domain and/or from across more jurisdictions, and use a broader array of evidence. In addition, longer timelines may be needed for topics that are politically or culturally sensitive topics. For example, a review focused on identifying best practices for implementing the United Nations Declaration on the Rights of Indigenous Peoples required a longer timeline for scope of the review (given that it included a review of the literature and key informant interviews with stakeholders in six countries) but also because of the time needed to build and maintain a partnership for conducting the review with several Indigenous groups.

Findings from these types of more complex syntheses can be used to generate an evolving framework based on emerging concepts from the literature and insights from key informants. In these instances, an initial framework may come existing theories or be derived from consultations such as when the review questions transdisciplinary and an overarching framework is unlikely to exist. As can be imagined, such approaches often address complex and broad questions. In addition to the example noted above, another example of this approach includes a rapid review conducted over several months that sought to inform efforts to create rapid-learning health systems in Canada. This involved a synthesis of the literature and key informant interviews to generate a definition of rapid-learning health systems that was relevant to Canada, hand searches of government and stakeholder websites of each of the 14 Canadian jurisdictions (the federal/national level, 10 provinces and three territories) to identify assets for a creating a rapid-learning health system, and 50 key informant interviews that were conducted across the country [25]. In addition, a common approach to rapid reviews which attracts little attention in the research literature is dissertations authored by postgraduate students bringing prior professional expertise. Re-analyzing existing systematic reviews of global literature allows them to tailor a new rapid synthesis focusing on their own professional interests. For instance, interpreting the findings of global evidence about women’s employment [26, 27] in light of a rapid review of studies in Spanish and Portuguese has provided valuable evidence for Brazil [28].

## Conclusion

The choice of approaches for conducting rapid reviews is intertwined with decisions about how to manage projects, the amount of work to be done, and the knowledge already available. In addition, the length of time required for conducting a review should also be considered through the lens of any political or cultural sensitivities that need to be addressed that may require a longer timeline to complete. Moreover, while the focus of this series is on complex questions related to health policy and system issues, the approaches we outline can be used to address complex social-system questions as well. Indeed, many complex policy questions often require considering both health- and social-systems issues given that such challenges often require transdisciplinary policy solutions. Given this, our guide offers support to help make these strategic decisions using the timeline provided as the starting point coupled with a guidance on how to engage in a consultative process as well as project management considerations that need to be taken into account in order to conduct rapid reviews.

## Supplementary Information

**Additional file 1.**

## Data Availability

The datasets used and/or analyzed during the current study are available from the corresponding author on reasonable request.

## Abbreviation

PICO: Population intervention controls and outcomes
