# Supplementary material for: Paper 3: Selecting rapid review methods for complex questions related to health policy and system issues
Source: Syst Rev. 2021 Oct 30;10:286. doi: 10.1186/s13643-021-01834-y (PMC8556903; doi:10.1186/s13643-021-01834-y)
Supplement: Supplementary file 1 — Additional file 1. [file 13643_2021_1834_MOESM1_ESM.docx]

Funding

This series was funded by the Alliance for Health Policy and Systems Research, an international partnership hosted by the World Health Organization, with support from the Norwegian Government Agency for Development Cooperation (Norad), the Swedish International Development Cooperation Agency (Sida) and the UK Department for International Development (DFID). Time to produce this manuscript was donated in kind by the authors’ respective organizations, but no other specific funding was received.
